# The Correlation Between International Medical Graduates’ Self-Study Habits and Their United States Medical Licensing Examination (USMLE) Step 1 and Step 2 Clinical Knowledge (CK) Scores

**DOI:** 10.7759/cureus.63798

**Published:** 2024-07-04

**Authors:** Maria Emilia C Garcia, Brinda K Navalgund

**Affiliations:** 1 Academic Department, Oceania University of Medicine, Edinburg, USA; 2 Physical Medicine and Rehabilitation Department, Indiana Regional Medical Center, Greensburg, USA

**Keywords:** us medical students, step 2 ck scores, step 1 scores, us residency, usmle step 2 ck, usmle step 1, non-us medical students, foreign medical graduates, imgs, international medical graduates

## Abstract

Introduction: To secure a residency in the United States, medical students must pass the United States Medical Licensing Examination (USMLE) Step 1 and Step 2 CK exams. This study examines the correlation between international medical graduates’ (IMGs) self-study habits and their USMLE scores.

Materials and methods: A retrospective study was conducted with 51 anonymous third- and fourth-year IMGs from Saint James Medical School, IL, United States. Participants completed an online survey about their study habits and USMLE Step 1 and Step 2 CK scores. All participants were undergoing clinical clerkships at South Texas Health Hospitals in McAllen, TX.

Results: The highest mean Step 1 scores were 211.3 for completing ≥7,000 questions, 222.2 for 91-120 days of study, 209.2 for 76-100% time on practice questions, 229.7 for 16-19 hours/day of study, and 228.0 for 51-75% group study. The highest mean Step 2 CK scores were 241.0 for completing ≥6,000 questions, 239.8 for <30 days of study, 238.8 for 76-100% time on practice questions, 239.0 for 16-19 hours/day of study, and 237.5 for 26-50% group study. No significant relationship was found between study habits and passing Step 1 scores (p>0.05), but moderate correlations were found for completing ≥4,000 questions and 61-90 days of study. No significant relationship was found between study habits and the national average Step 2 CK score, but a strong correlation was found for 25-50% time on practice questions.

Conclusion: While some study habits correlate with higher scores, no significant relationship was found between specific study habits and passing Step 1 or achieving the national average Step 2 CK score.

## Introduction

The United States Medical Licensing Examinations (USMLEs) consist of three steps, each of which must be successfully passed for medical students to obtain a Doctor of Medicine (MD) degree [1]. All three examinations are computer-based, but for the purposes of this research study, we focus on the specifics of USMLE Step 1 and Step 2 CK (clinical knowledge). USMLE Step 1 assesses the fundamental biomedical science principles underlying modern medical practice [2], while USMLE Step 2 CK covers various medical specialties, demanding a comprehensive understanding of clinical concepts, diagnosis, and management relevant to patient care under supervision [3].

Most medical students take Step 1 just before starting their clinical clerkships, typically after their second year in a traditional four-year program [4]. Contrary to its initial purpose as a licensing requirement, residency programs often utilize USMLE Step 1 scores as a screening tool for applicants [5-7]. In fact, it was once considered the most influential factor in resident selection nationwide [6]. However, this changed when Step 1 transitioned from a three-digit score to a pass/fail system in January 2022, aiming to alleviate the burden of exam preparation for medical students and foster a more holistic residency application process [5]. While some view this change positively, others question whether the pressure now shifts to achieving a competitive Step 2 CK score [8].

For competitive medical specialties (those with the highest ratios of applicants to available residency positions), a significantly higher Step 2 CK score is often required to secure a US residency position [9]. Moreover, international medical graduates (IMGs) must attain higher Step 2 CK scores compared to their American counterparts to have an equivalent chance of matching into a US residency position. Many programs even disregard other aspects of an applicant’s profile if their Step 2 CK score falls below a certain threshold [9].

Despite the significance of USMLE scores for residency and specialty selection, little is known about predictors of both passing and competitive scores. Existing literature primarily focuses on prior performance measures, such as Medical College Admission Test (MCAT) scores or class grades, as predictors for USMLE performance [10,11], showing only modest correlations. Previous investigations into specific study habits for Step 1, such as hours spent studying per week and optimal testing dates, have yielded inconclusive results [12,13]. These studies also did not evaluate additional study habits proven to be beneficial outside of medical training, such as practice question utilization [14] or group studying [15-18]. Additionally, these studies focused solely on Step 1 scores among primarily US medical students, without considering benchmarks for non-US medical students.

In spite of recent increases in the number of US medical graduates, IMGs constitute an important part of the US healthcare system and will continue to be needed to meet the nation's demands for medical services [19,20]. Due to differences in culture, education, and local healthcare systems, IMG applicants and residents and the institutions preparing them for their professional careers experience financial, social, and political challenges, as well as personal, emotional, and financial hardships [4].

The primary aim of this study is to further characterize the study habits reported by non-US medical students for USMLE Step 1 and/or Step 2 CK and determine whether any of these habits correlate with both passing Step 1 scores and competitive Step 2 CK scores. The study also seeks to investigate study habits not previously explored in medical training, such as the number of practice questions completed, daily study hours during dedicated periods, and the percentage of time spent in group settings during dedicated study periods. Furthermore, the author hypothesizes that completing at least 4,000 practice questions, devoting at least 61-90 days to dedicated studying, devoting at least 25-50% of time to doing practice questions alone, studying at least 8-11 hours/day, and studying at least 1-25% of time in a group setting are associated with a passing USMLE Step 1 score of 196 or higher (or 194 or higher if taken prior to January 2022) and a USMLE Step 2 CK score of 245 or higher, which is the current national average as of 2024.

The USMLEs are high-stakes examinations that can induce significant anxiety in test takers [21]. Any insights into how students prepare for these exams would be invaluable, especially those at risk of failure.

## Materials and methods

In this retrospective cross-sectional study, 51 anonymous third- and fourth-year IMGs from Saint James Medical School (SJMS) were analyzed based on their self-reported study habits and USMLE score(s). The inclusion criteria included having already taken either USMLE Step 1 and/or Step 2 CK, having already obtained scores for USMLE Step 1 and/or Step 2 CK, and having taken either exam no earlier than January 1, 2018, as this was when a minimum passing score of 194 went into effect. All participants who did not meet these criteria were excluded from the study.

During the time of the study, all participants were currently undergoing clinical clerkships in McAllen, TX. SJMS is accredited by the Accreditation Commission on Colleges of Medicine (ACCM). Specifically, SJMS requires that their students take the USMLE Step 1 within four months of completing the Basic Science program [22]. Furthermore, all students must receive a passing Step 1 score in order to move forward with their clinical rotations, making the third- and fourth-year students eligible participants in this study as they automatically meet the inclusion criteria stated above.

Participation in this study was completely voluntary. Students who chose to participate in the study were sent an online Google Survey link via text message, which consisted of a 9-18-question anonymous survey. Participants were asked to complete either section 1 alone (questions 1-9) if they have only taken USMLE Step 1, or both section 1 and section 2 (questions 1-18) if they have taken both USMLE Step 1 and Step 2 CK. Table 1 shows the format of the online survey administered to the participants, which included both multiple-choice and open-ended questions. No replications or changes to the survey were allowed once a participant submitted their survey.

**Table 1:** Sample survey used in this study

Questions	Response options
Have you taken USMLE Step 1?	A. Yes
	B. No
How many attempts did it take you to pass USMLE Step 1?	A. One attempt
	B. Two attempts
	C. Three attempts
	D. Four or more attempts
Please estimate the date of your most recent USMLE Step 1 exam?	(Open-ended responses)
What was your most recent USMLE Step 1 score? If no 3-digit score was given, please answer with either "pass" or "fail"	(Open-ended responses)
Please estimate the number of practice questions completed throughout the course of your USMLE Step 1 preparation.	A. <500 questions
	B. ~1000 questions
	C. ~2000 questions
	D. ~3000 questions
	E. ~4000 questions
	F. ~5000 questions
	G. ~6000 questions
	H. ~7000 questions
	I. >8000 questions
Please estimate the number of days devoted to your dedicated studying for USMLE Step 1.	A. <30 days
	B. 30-60 days
	C. 61-90 days
	D. 91-120 days
	E. 121-150 days
	F. 151-180 days
	G. >180 days
Please estimate the percentage of time you allocated doing USMLE step 1 practice questions during your dedicated studying.	A. <25%
	B. 25-50%
	C. 51-75%
	D. 76-100%
	E. I did not do any practice questions
Please estimate the average number of hours per day you spent studying during your dedicated studying for USMLE Step 1.	A. 0-3 hours
	B. 4-5 hours
	C. 8-11 hours
	D. 12-15 hours
	E. 16-19 hours
Please estimate the percentage of time you spent studying for USMLE Step 1 in a group setting.	A. 0%
	B. 1-25%
	C. 26-50%
	D. 51-75%
	E. 76-100%
**If you have not taken USMLE Step 2 CK, please submit the survey now.	
Have you taken USMLE Step 2 CK?	A. Yes
	B. No
How many attempts did it take you to pass USMLE Step 2 CK?	A. One attempt
	B. Two attempts
	C. Three attempts
	D. Four or more attempts
Please estimate the date of your most recent USMLE Step 2 CK exam.	(Open-ended responses)
What was your most recent USMLE Step 2 CK score?	(Open-ended responses)
Please estimate the number of practice questions completed throughout the course of your USMLE Step 2 CK preparation.	A. <500 questions
	B. ~1000 questions
	C. ~2000 questions
	D. ~3000 questions
	E. ~4000 questions
	F. ~5000 questions
	G. ~6000 questions
	H. ~7000 questions
	I. >= 8000 questions
Please estimate the number of days devoted to your dedicated studying for USMLE Step 2 CK.	A. <30 days
	B. 30-60 days
	C. 61-90 days
	D. 91-120 days
	E. 121-150 days
	F. 151-180 days
	G. >180 days
Please estimate the percentage of time you allocated doing USMLE step 2 CK practice questions during your dedicated studying.	A. <25%
	B. 25-50%
	C. 51-75%
	D. 76-100%
	E. I did not do any practice questions
Please estimate the average number of hours per day you spent studying during your dedicated studying for USMLE Step 2 CK.	A. 0-3 hours
	B. 4-5 hours
	C. 8-11 hours
	D. 12-15 hours
	E. 16-19 hours
Please estimate the percentage of time you spent studying for USMLE Step 2 CK in a group setting.	A. 0%
	B. 1-25%
	C. 26-50%
	D. 51-75%
	E. 76-100%

The minimum target sample size was 50, which was to be collected in a span of two months (or earlier if the target sample size had been reached). Ultimately, a sample size of 51 was reached within one month. Although this is a small sample size, this research study can offer preliminary insights into the self-study habits and USMLE scores of IMG students, which can ultimately guide more extensive research and help formulate hypotheses for larger studies. Additionally, since IMG students may have different experiences and challenges compared to US medical graduates, this survey can shed light on unique aspects of their preparation and performance. Overall, while a small sample size limits the generalizability of the findings, the insights gained can still be beneficial for the larger population in guiding future research, informing educational policy, and providing initial data that highlights important areas in this untouched topic of research for IMGs. 

The study was approved by the Ethics Committee at the Oceania University of Medicine (HREC24_022). Informed consent was obtained from the student participants, and anonymity was guaranteed. There was no cost associated with the study.

Steps of data analysis and software used

The dataset used for this analysis was provided in a CSV file (usmle_data_cleaned.csv) and analyzed using Python in a Jupyter Notebook (USMLE_Analysis.ipynb). All software and libraries used include the following: (1) Python: p programming language used for data analysis; (2) Jupyter Notebook: an interactive environment for data analysis and visualization; (3) Pandas: library for data manipulation and analysis; (4) Scikit-learn: library for machine learning, used for logistic regression; (5) SciPy: library for statistical analysis, including the chi-square test; and (6) Openpyxl: library for writing results to Excel (Microsoft® Corp., Redmond, WA) files.

The dataset was loaded using Pandas. Missing values were handled by dropping rows with incomplete data to ensure the accuracy of the analysis. Binary variables were created to categorize the following study behaviors: (1) High_Score: whether the score was greater than or equal to a specified threshold (e.g., 196 for Step 1 and 245 for Step 2); (2) Studied_4000_Questions: whether the number of practice questions was greater than or equal to 4,000; (3) Many_Days_Studying: whether the number of study days was greater than or equal to 110; (4) High_Percent_Time_Studying: whether the percentage of study time was greater than or equal to 50%; (6) Many_Hours_Per_Day: whether the number of study hours per day was greater than or equal to 11.; and (7) High_Study_Group_Percent: whether the percentage of study time spent in groups was greater than or equal to 25%.

Logistic regression models were fitted to predict high scores based on the binary study behavior variables. Data were split into training and testing sets (80-20 split). Model performance was evaluated using a confusion matrix, classification report, and model coefficients. Lastly, chi-square tests were performed to determine if there was a significant association between each study behavior and achieving a high score.

In the logistic regression (Step 1) model, there was an accuracy of 91% but only predicted the positive class correctly. Positive correlation with studying 4,000+ questions and many days of studying. Negative correlation with a high percentage of study time, many hours per day, and high study group percentage.

In the logistic regression (Step 2) model, results revealed relationships between study behaviors and high scores. In the chi-square analysis, no significant associations were found between individual study behaviors and high scores in both Step 1 and Step 2 analyses.

## Results

The survey was sent to 75 students, and 51 students responded (68% response rate). Data collection started on March 28, 2024, and ended on May 18, 2024. All participants were currently undergoing their clinical clerkships; had at least taken their USMLE Step 1 examination on or after January 1, 2018; and had already obtained their USMLE step score(s). No respondents were excluded.

Tables 2-3 represent the results of survey respondent characteristics and their USMLE Step 1 scores. For analysis purposes and to avoid overestimation of the data, all students who responded with a “Pass” as their USMLE Step 1 score were given a three-digit score of 196 as this value represents the minimum passing score threshold, and all scores above this threshold are considered passing. No students responded with a “Fail,” so a score threshold was not needed in this scenario.

**Table 2:** Mean scores of self-reported study habits for USMLE Step 1 This shows the mean USMLE Step 1 scores for each variable tested on the survey.

Variable	Sample (N)	Mean score
Number of practice questions completed	51	N/A
<500 questions	1	196.0
1000 questions	0	0.0
2000 questions	6	205.8
3000 questions	10	210.5
4000 questions	15	209.9
5000 questions	4	201.5
6000 questions	8	204.3
7000 questions	4	211.3
>=8000 questions	3	211.0
Number of days devoted to dedicated studying	51	N/A
<30 days	3	197.0
30-60 days	9	214.6
61-90 days	10	208.2
91-120 days	5	222.2
121-150 days	3	196.0
151-180 days	2	199.0
>180 days	19	205.4
Percentage of time devoted to practice questions alone	51	N/A
<25%	3	207.7
25-50%	4	197.0
51-75%	17	208.5
76-100%	27	209.2
I did not do any practice question	0	0.0
Hours per day devoted to dedicated studying	51	N/A
0-3 hours	5	203.8
4-5 hours	7	204.7
8-11 hours	29	206.9
12-15 hours	7	208.7
16-19 hours	3	229.7
Percentage of time studying in a group setting	51	N/A
0%	21	203.0
1-25%	27	209.6
26-50%%	2	225.5
51-75%	1	228.0
76-100%	0	0.0

**Table 3:** P-value and correlation between the USMLE Step 1 score and variables under investigation This shows the mean USMLE Step 1 examination scores and their standard deviations for all variables under investigation.

Variable	Sample (N)	Mean (SD)	Chi^2^ test	p-value	Correlation coefficient (r)
USMLE Step 1 score	51	207.8 (17.3)	N/A	N/A	N/A
Number of practice questions completed	51	4362.7 (1786.3)	0	1	0.533
Number of days devoted to dedicated studying	51	116.6 (58.2)	0	1	0.407
Percentage of time devoted to practice questions alone	51	68.5 (23.9)	0	1	-0.141
Hours per day devoted to dedicated studying	51	10.2 (3.7)	0	1	-0.228
Percentage of time studying in a group setting	51	16.7 (16.3)	0.225	0.635	-0.291

In Table 2, the results revealed that the highest mean examination scores were 211.3, 211.0, and 210.5 for completing ~7,000 questions, >=8,000, and ~3,000 questions, respectively. The highest mean examination scores were 222.2, 214.6, and 208.2 for devoting between 91-120 days, 30-60 days, and 61-190 days, respectively, to dedicated studying. The highest mean examination scores were 209.2, 208.5, and 207.7 for devoting between 76% and 100%, 51% and 75%, and <25% of the time, respectively, to doing practice questions alone. The highest mean examination scores were 229.7, 208.7, and 206.9 for devoting between 16 and 19 hours/day, 12 and 15 hours/day, and 8 and 11 hours/day, respectively, to dedicated studying. Lastly, the high mean examination scores were 228.0, 225.5, and 209.6 for devoting between 51% and 75%, 26% and 50%, and 1% and 25% of the time, respectively, to studying in a group setting.

In Table 3, the p-value was calculated using a chi-square analysis (chi^2^ test). No significant relationship was found between all five variables under investigation and achieving a minimum passing score of 196 (p>0.05). The correlation coefficient (r) was calculated using Pearson correlation. A moderate positive correlation was found between completing 4,000 or more practice questions and achieving a minimum passing score of 196 (r=0.533). A moderate positive correlation was also found between devoting at least 61-90 days to dedicated studying and achieving a minimum passing score of 196 (0.407); however, no correlation was found with the percentage of time devoted to practicing questions alone, hours per day devoted to dedicated studying, and percentage of time studying in a group setting.

Tables 4-5 represent the results of survey respondent characteristics and their USMLE step 2 CK scores. The data were analyzed similarly to Tables 2-3; however, the exact three-digit USMLE Step 2 CK score provided by each respondent was used. No minimum score threshold was needed.

**Table 4:** Mean scores of self-reported study habits for USMLE Step 2 CK This shows the mean USMLE Step 2 CK scores for each variable tested on the survey.

Variable	Sample (N)	Mean score
Number of practice questions completed	36	N/A
<500 questions	1	234.0
1000 questions	0	0.0
2000 questions	4	231.6
3000 questions	11	236.7 (13.9)
4000 questions	8	234.1
5000 questions	8	231.4
6000 questions	2	241.0
7000 questions	0	0.0
>=8000 questions	2	225.0
Number of days devoted to dedicated studying	36	N/A
<30 days	4	239.8
30-60 days	11	236.5
61-90 days	8	234.6
91-120 days	6	228.8
121-150 days	1	219.0
151-180 days	3	227.7
>180 days	3	236.3
Percentage of time devoted to practice questions alone	36	N/A
<25%	3	227.3
25-50%	4	225.0
51-75%	11	230.9
76-100%	18	238.8
I did not do any practice question	0	0.0
Hours per day devoted to dedicated studying	36	N/A
0-3 hours	3	227.3
4-5 hours	7	238.7
8-11 hours	18	232.8
12-15 hours	7	234.1
16-19 hours	1	239.0
Percentage of time studying in a group setting	36	N/A
0%	17	233.9
1-25%	16	234.2
26-50%	2	237.5
51-75%	1	223.0
76-100%	0	0.0

**Table 5:** P-value and correlation between the USMLE Step 2 CK score and variables under investigation This shows the mean USMLE Step 2 CK examination scores and their standard deviations for all variables under investigation.

Variable	Sample (N)	Mean (SD)	Chi^2^ test	p-value	Correlation coefficient (r)
USMLE Step 2 CK score	36	233.9 (11.0)			
Number of practice questions completed	36	3930.6 (1577.3)	0	1	-0.218
Number of days devoted to dedicated studying	36	85.1 (49.9)	0.7	0.403	-0.849
Percentage of time devoted to practice questions alone	36	66.3 (25.1)	2.48	0.115	0.858
Hours per day devoted to dedicated studying	36	10.1 (3.9)	0.002	0.963	-0.764
Percentage of time studying in a group setting	36	16.0 (18.1)	0	1	-0.088

In Table 4, the results revealed that the highest mean examination scores were 241.0, 236.7, and 234.1 for completing ~6,000 questions, 3,000, and ~4,000 questions, respectively. The highest mean examination scores were 239.8, 236.5, and 236.3 for devoting <30 days, between 30 and 60 days, and >180 days, respectively, to dedicated studying. The highest mean examination scores were 238.8, 230.9, and 227.3 for devoting between 76% and 100%, 51% and 75%, and <25% of the time, respectively, to doing practice questions alone. The highest mean examination scores were 239.0, 238.7, and 234.1 for devoting between 16 and 19 hours/day, four and five hours/day, and 12 and 15 hours/day, respectively, to dedicated studying. Lastly, the high mean examination scores were 237.5, 234.2, and 233.9 for devoting between 26% and 50%, 1% and 25%, and 0% of the time, respectively, to studying in a group setting.

In Table 5, the p-value was calculated using a chi-square analysis (chi^2^ test). No significant relationship was found between all five variables under investigation and achieving a minimum score of 245, which is the current national average score (p>0.05). The correlation coefficient (r) was calculated using Pearson correlation. A strong positive correlation was found between devoting at least 25-50% of the time to doing practice questions alone and achieving a minimum score of 245 (r=0.858); however, no correlation was found with the number of practice questions completed, number of days devoted to dedicated studying, hours per day devoted to dedicated studying, and percentage of time studying in a group setting.

## Discussion

The USMLEs are high-stakes examinations that can induce significant anxiety in test takers [21]; therefore, any insights into how students prepare for these exams would be invaluable, especially those at risk of failure. This retrospective research survey study analyzed the correlation between 51 IMGs’ reported self-study habits and their USMLE step scores from 2018 to 2024. The results revealed that there was a moderate correlation between completing at least 4,000 questions (r=0.533) and devoting at least 61-90 days to dedicated studying (r=0.407) and achieving a minimum passing USMLE Step 1 score of 196 (Table 3). A strong positive correlation was also found between devoting at least 25-50% of the time to doing practice questions alone and achieving a minimum USMLE Step 2 CK score of 245 (Table 5). However, no significant difference was found between achieving a minimum passing Step 1 score and a minimum national average Step 2 CK score and completing at least 4,000 practice questions, devoting at least 61-90 days to dedicated studying, devoting at least 25-50% of the time to doing practice questions alone, studying at least 8-11 hours/day, and studying at least 1-25% of the time in a group setting (p>0.05). Therefore, we fail to reject the null hypothesis.

In a similar study focusing on self-study habits and USMLE Step 1 scores of 256 third- and fourth-year medical students at Tulane University [23], results revealed that respondents who estimated they completed a greater number of practice questions had higher Step 1 scores. However, the students who reported spending a greater portion of their total study time on practice questions alone did not have significantly higher scores. When comparing this to our results, we found that IMGs who estimated they completed a greater number of practice questions had higher mean Step 1 scores (Table 2), but results varied with mean Step 2 CK scores (Table 4). Contrarily, IMGs who reported spending a greater portion of their total study time on practice questions alone had higher mean Step 2 CK scores (Table 4), but results varied with mean Step 1 scores (Table 2).

When comparing Step 1 scores between the US medical students from Tulane University and those of IMG students from SJMS, similar upward trends were observed [23]. For both studies, the highest mean scores were seen with completing >8,000 practice questions (247 and 211, respectively) and devoting at least 16-19 hours/day in the dedicated studying period (233.3 and 229.7, respectively). However, the SJMS students who reported completing a greater amount of practice questions did not have significantly higher mean scores compared to that seen in students from Tulane University. It is important to note that this difference could be the result of a significantly smaller sample size of SJMS students compared to that of Tulane University, as well as the substitution of all reported “Pass” scores as 196.

Now that USMLE Step 1 has changed from a three-digit score to a pass/fail system, future studies are indicated to determine what USMLE Step 2 CK scores are needed for IMGs to obtain the same competitive residencies as their US counterparts. By doing so, this particular demographic of medical students will be given more insight as to what three-digit range they should be scoring in to at least secure an interview at these competitive programs.

Limitations

This study has several limitations. One limitation previously addressed in the study is the small sample size, which can limit the ability to detect statistically significant differences or correlations, ultimately leading to a higher likelihood of type II errors. This can also result in greater variability and less reliable estimates of the true population parameters as individual differences can have a more pronounced effect on the results in a small sample. Another limitation is recall bias as most of the research participants had to recall their study habits from more than a year ago, which can lead to a tendency to over-/underestimate study habits based on the final examination score they received. A third limitation is data generalization of the USMLE Step 1 scores as all students who received a “Pass” were given a numerical score of 196, which could underestimate and skew the data. Finally, since the data were collected by survey, it cannot demonstrate causality nor can it reliably predict future performance. One strength was that the sample collected from this one Caribbean medical school comprised a fair representation of IMGs as there was a mixture of both US residents and foreign residents who participated in the study. Overall, this study focused on an untouched topic of research for IMGs; therefore, any insights gained can still be beneficial to the larger population in guiding future research, informing educational policy, and providing initial data that highlight important areas for further investigation.

## Conclusions

In conclusion, this study aimed to investigate the correlation between self-reported study habits and USMLE Step 1 and Step 2 CK scores among IMGs. The results revealed moderate-to-strong positive correlations between certain study habits and achieving passing or competitive scores. However, no significant relationship was found between achieving passing Step 1 scores or the national average Step 2 CK score and completing at least 4,000 practice questions, devoting a specific number of days to dedicated studying, allocating a certain percentage of time to doing practice questions alone, studying a specific number of hours per day, and studying in a group setting. While the study provides valuable insights into the study habits of IMGs, there are limitations such as small sample size, recall bias, and data generalization. Future studies are needed to determine the Step 2 CK scores required for IMGs to secure competitive residencies, especially in the context of the recent change in the Step 1 scoring system to pass/fail.
